# What factors empower general practitioners for early cancer diagnosis? A 20-country European Delphi Study

**DOI:** 10.1017/S1463423622000652

**Published:** 2022-11-25

**Authors:** George Tzanis, Michael Harris, Mette Brekke, Mercè Marzo-Castillejo, Saliha Serap Cifcili, Wojciech Wawrzynek, Maria Flamm, Nicola Buono, Bernadett Márkus, Galia Zacay, Ilze Skuja, Zlata Ozvacic Adzic, Mihai Iacob, Radost Asenova, Davorina Petek, Krzysztof Buczkowski, Pamela Curtis, Liina Pilv-Toom, Robert Hoffman, Emmanouil Smyrnakis

**Affiliations:** 1 Laboratory of Primary Health Care, General Practice and Health Services Research, Aristotle University of Thessaloniki, Thessaloniki, Greece; 2 College of Medicine & Health, University of Exeter, Exeter, UK; 3 Institute of Primary Health Care Bern (BIHAM), University of Bern, Bern, Switzerland; 4 General Practice Research Unit, University of Oslo, Oslo, Norway; 5 Cancer Research Group in Primary Health Care, Fundació Institut Universitari per a la Recerca a l’Atenció Primària de Salut Jordi Gol i Gurina (IDIAPJGol), Barcelona, Spain; 6 Unitat de Suport a la Recerca Metropolitana Sud, Direcció d’Atenció Primària Costa de Ponent, ICS, Barcelona, Spain; 7 Family Medicine Department, School of Medicine, Marmara University, Istanbul, Turkey; 8 The Medical Centre, Roscommon, Ireland; 9 Paracelsus Medical University; Institute of General Practice, Salzburg, Austria; 10 Family Medicine and Preventive Medicine, Center for Public Health and Healthcare Research, Salzburg, Austria; 11 National Society of Medical Education in General Practice (SNAMID), Caserta, Italy; 12 Department of Family Medicine, Semmelweis University, Budapest, Hungary; 13 Department of family medicine, Sackler Faculty of Medicine, University of Tel Aviv, Tel Aviv, Israel; 14 Department of family medicine, Meuhedet Health Maintenance Organization, Israel; 15 Department of Family Medicine, Riga Stradins University, Riga, Latvia; 16 School of Medicine, Department of Family Medicine, University of Zagreb, Zagreb, Croatia; 17 Zagreb-Centar Health Center, Zagreb, Croatia; 18 EUVEKUS, Timis Society of Family Medicine, Timisoara, Romania; 19 Department of Urology and General Practice, Faculty of Medicine, Medical University of Plovdiv, Plovdiv, Bulgaria; 20 Department of Family medicine, Faculty of Medicine, University of Ljubljana, Ljubljana, Slovenia; 21 Department of Family Medicine, Nicolaus Copernicus University, Torun, Poland; 22 Close Farm Surgery, UK; 23 Institute of Family Medicine and Public Health, University of Tartu, Tartu, Estonia

**Keywords:** cancer, Delphi method, empowerment, general practitioners, Primary Health Care

## Abstract

**Background:**

Some symptoms are recognised as red flags for cancer, causing the General Practitioner (GP) to refer the patient for investigation without delay. However, many early symptoms of cancer are vague and unspecific, and in these cases, a delay in referral risks a diagnosis of cancer that is too late. Empowering GPs in their management of patients that may have cancer is likely to lead to more timely cancer diagnoses.

**Aim:**

To identify the factors that affect European GPs’ empowerment in making an early diagnosis of cancer.

**Methods:**

This was a Delphi study involving GPs in 20 European countries. We presented GPs with 52 statements representing factors that could empower GPs to increase the number of early cancer diagnoses. Over three Delphi rounds, we asked GPs to indicate the clinical relevance of each statement on a Likert scale.

The final list of statements indicated those that were considered by consensus to be the most relevant.

**Results:**

In total, 53 GPs from 20 European countries completed the Delphi process, out of the 68 GPs who completed round one. Twelve statements satisfied the pre-defined criteria for relevance. Five of the statements related to screening and four to the primary/secondary care interface. The other selected statements concerned information technology (IT) and GPs’ working conditions. Statements relating to training, skills and working efficiency were not considered priority areas.

**Conclusion:**

GPs consider that system factors relating to screening, the primary-secondary care interface, IT and their working conditions are key to enhancing their empowerment in patients that could have cancer. These findings provide the basis for seeking actions and policies that will support GPs in their efforts to achieve timely cancer diagnosis.

## Background

Every day, patients present to their General Practitioners (GPs) with symptoms which could be due to cancer (Hamilton, 2009). Some symptoms are recognised as red flags for cancer, resulting in the GP referring the patient for further diagnostic tests without delay. However, many early symptoms of cancer are vague and unspecific, and are similar to those of benign or self-limiting conditions (Foot and Harrison, 2011). The GP faces a difficult balancing act between investigating more patients, which puts a strain on healthcare resources, and investigating less, so risking a delayed diagnosis of cancer (Nicholson, *et al.*, 2016). Several factors may influence GPs’ actions, including medical competence, workload and system factors like access to investigations and referral pathways (Harris, *et al.*, 2018; Harris, *et al.*, 2019), mirroring different levels of GPs’ empowerment.

Empowerment has been defined as ‘the process of gaining freedom and power to do what you want or to control what happens to you’ (Cambridge Dictionary, 2021). Roller describes empowerment as a product of three dimensions: autonomy (peoples’ perception of the level of freedom and personal control in the performance of their job), participation (the degree to which they feel they contribute to their organisation’s administrative or strategic decisions) and responsibility (the level of concern, care and commitment that they bring to a task or position) (Roller, 1998). One aspect of empowerment is structural empowerment, which can be described as the structures within an organisation that empower clinicians, for example, to practice in a professional and autonomous manner and achieve clinical excellence and professional fulfilment. According to Kanter, power is defined as the ‘ability to mobilise resources to get things done’ (Kanter, 1993). Kanter believes that access to four ‘sources’ or ‘lines’, namely information, support, resources and opportunities to learn and grow, are key determinants of empowerment within an organisation. It has been demonstrated that there is a link between empowering work settings and outcomes like job satisfaction, organisational commitment and effectiveness (Orgambídez-Ramos and Borrego-Alés, 2014). Empowerment is seen as being critical to clinicians’ ability to effect change in the healthcare system and society (Falk-Rafael AR, 2004).

There are few studies in relation to what empowers doctors (Verbeten, 2007; Mesko and Győrffy, 2019; Yakes *et al.*, 2020). Those studies have focused on the shifting power from healthcare providers towards employers, and how managed care regulation affects providers (Brown and Eagan, 2004). Lately, the effect of the challenges and demands of digital health care on doctors’ empowerment have attracted interest (Mesko and Győrffy, 2019). The increasing interest in doctors’ health and well-being indicates the need to consider their perspectives on what empowers them in terms of job satisfaction. (Yakes *et al.*, 2020). Most studies on empowerment in healthcare settings have focused on the nursing profession (Irvine *et al.*, 1999; Fullam *et al.*, 1998; Wong and Laschinger, 2013; Orgambídez-Ramos and Borrego-Alés, 2014; Trus *et al.*, 2019). One study showed that the more nurses perceive they have access to workplace empowerment structures, the more they feel satisfied with their work and report higher performance (Wong and Laschinger, 2013).

Doctors who are empowered in their care of patients are less likely to face obstacles in the diagnostic pathway and more likely to be enabled to make timely diagnoses. This study therefore aims to identify factors that European GPs feel empower them to make a timely diagnosis in patients with symptoms which could be due to cancer.

## Methodology

This multicentre study used the Delphi technique, as this method allows us to collect and achieve consensus on panellists’ opinions. It is a structured communication process, where a panel of experts answers questions to which there are no scientifically proven correct answers (Linstone *et al.*, 1975; Linstone, 1985; Boulkedid *et al.*, 2011). It enables anonymous systematic refinement of expert opinions, with the aim of arriving at a combined or consensus position. The assumption is that the judgement of a group of experts, the ‘Delphi panellists’, will be more valid than individual judgements. Consensus is reached through consecutive rounds in which panellists are invited to modify their answers as a result of seeing the responses made by the panel in previous rounds.

Our study was organised through the Örenäs Research Group (ÖRG), a group of primary care researchers in 30 European countries that studies the primary care factors that relate to cancer survival. A core project group of eight members planned the study.

### Outcome measure

The primary outcome was the identification of empowerment factor statements with the most agreement among GP panellists that they were clinically relevant in a primary care setting.

### Generation of the statements

During the preliminary phase, the core project group members each wrote a list of factors that they believed affect GPs’ empowerment in making an early cancer diagnosis. The resulting 97 factors were collated and put in alphabetical order in the form of 77 statements. Each statement started with the following phrase:

*‘GPs would be empowered to increase the number of early cancer diagnoses by…’.*

In a pilot round, all other ÖRG members (*n* = 76) were invited to study the list of statements and suggest additions. Proposed additions were reviewed by the core project group. For each statement, ÖRG members were asked to evaluate its clinical relevance in a primary care setting, using a Likert scale ranging from 1 (no clinical relevance) to 9 (highly clinically relevant), to comment on the statements, and suggest any statements that they thought were missing. We received responses from 40 ÖRG members in 23 European countries. Respondents made 121 comments and suggestions: where these suggested that any statements were ambiguous or unclear, we changed the wording of the statements. No new statements arose as a result of this process.

The core project group had decided that the number of statements included in the final list should be around 50, to achieve a balance between having enough statements and maintaining panellists’ interest throughout the Delphi study.

After analysing the results and omitting the statements with the lowest Likert scores, we produced a final list of 52 statements. The project coordinators grouped these statements according to their content. The number and names of the groups, and the distribution of the statements in the groups, were discussed and agreed upon by the core project group. For example, all the statements related to cancer screening were placed in one group, whereas those related to tests for cancer formed another group. The resulting 14 groups of statements are given in Appendix 1.

### Recruitment

All ÖRG members were sent a copy of the study protocol and invited to recruit panellists in their countries (as ‘Local Leads’). Where there was more than one member in a country, they were asked to agree who would be the Local Lead. The project coordinators produced a model invitation email for possible panellists that each Local Lead could adapt according to their needs. Local Leads were asked to approach seven potential Delphi panellists and ask for consent to give their names and email addresses to the project coordinators.

The project coordinators then contacted these potential Delphi panellists by email (five for each country, with two in reserve) with information about the study, a Participant Information Sheet, an invitation to join the study and a link to the survey if they decided to take part. Possible panellists from the reserve lists were invited only if we did not receive five responses from that country.

The recruitment criteria that the Local Leads were asked to use to select the Delphi panellists are shown in Table 1.

**Table 1.** The Delphi panellists’ recruitment criteria

*Mandatory*
Panellists must have qualified as GPs.
At least 3 panellists from each country have to be working as GPs or to have worked as GPs in the previous two years.
Any panellists who are not current or recent GPs must be working as GP academics with a special interest in cancer in primary care.
*Preferable*
At least 2 should be female, 1 male.
At least 2 should have less than 15 years of working experience since graduation.
At least 2 should work in urban, and 2 in rural, areas.

### Delphi rounds

The Delphi process consisted of 3 rounds:

- Round 1. Panellists were presented with the list of statements, which were organised in five randomly ordered survey pages. For each statement, the panellists were asked to score the statements on a Likert scale from 1 (no clinical relevance in a primary care setting) to 9 (highly clinically relevant). Panellists who had not replied or completed the survey were sent two reminders. The core project group calculated mean scores with their standard deviations (SDs) for each statement.
- *Round 2*. Panellists were shown the list of statements in descending order of the first round’s mean Likert scores, accompanied by their SDs, and were asked to consider these while completing the Likert scales again. After two reminders to non-responders, the project coordinators once more calculated mean scores and SDs for each statement.
- *Round 3*. Panellists were shown the list of statements in descending order of the second round’s mean Likert scores, accompanied by their corresponding SDs. They were asked to consider these while completing the Likert scales for a final time. The Delphi data collection ended after we had sent two reminders to non-responders.

### Data collection

The data were collected with a web-based survey platform (SurveyMonkey Inc.). Survey links were provided by the project coordinators. The survey language was English.

The first survey page described the aim of the study, the role of the Örenäs Research Group and the process of the Delphi study. A web link led to further details, including information about data protection. There was a statement that participation was entirely voluntary and that the panellists could withdraw at any time.

The second page asked for demographic details: country, whether worked as a GP in the last two years, whether working as a GP academic, sex, years of working experience since graduation and working area (rural, urban or mixed).

Next, five survey pages presented the 52 statements. Each page presented three statement groups (except for one page with only two statement groups). To reduce the risk of order-effects bias (Israel and Taylor, 1990), we constructed five versions of the survey, each with a different URL and a different order of the five statement pages. Within the statement groups, the statements were presented in alphabetical order. In each country, every panellist received a different URL link.

### Statistical analysis

The demographic characteristics of the respondents were explored using descriptive statistics.

For each statement, the degree of consensus was based on the mean Likert score and the corresponding standard deviation (SD), as a lower SD indicates increased concordance between panellists’ responses. To select statements with substantial consensus, we pre-defined the threshold for inclusion in the final list of statements as the mean Likert score minus one SD ≥ 5 (after Nyborg *et al.*, 2015).

**Figure 1.**
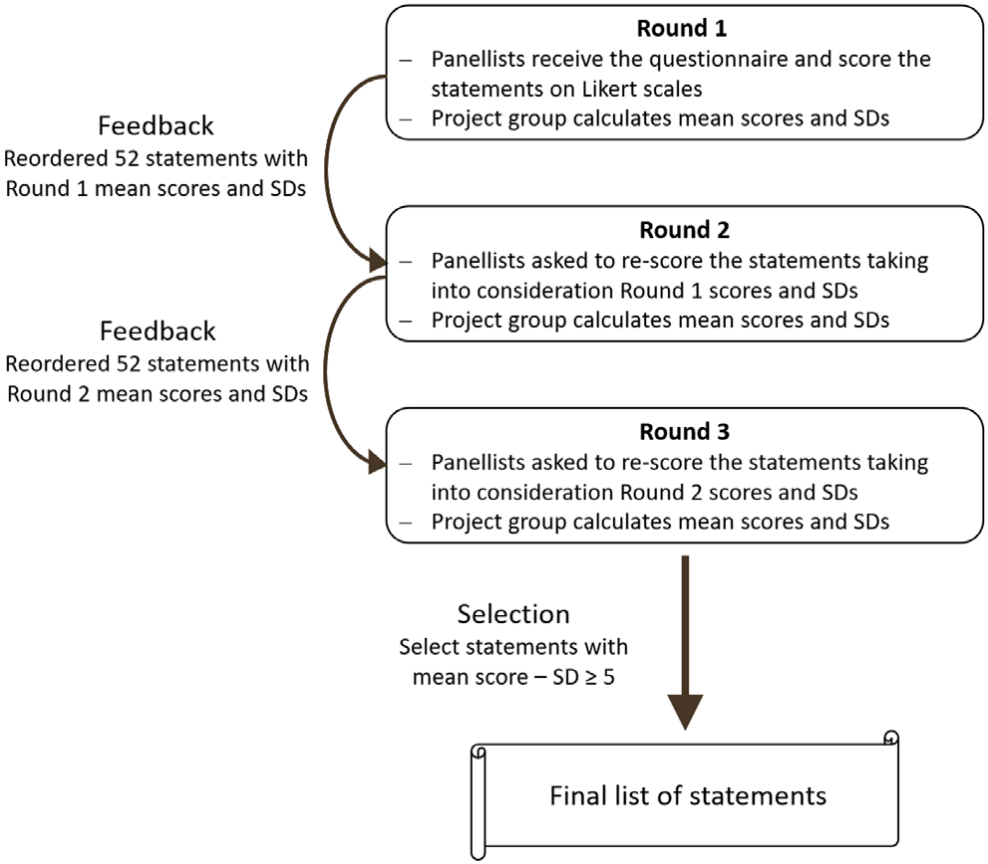
The Delphi study process.

Figure 1 summarises the process of the study.

## Results

**Figure 2.**
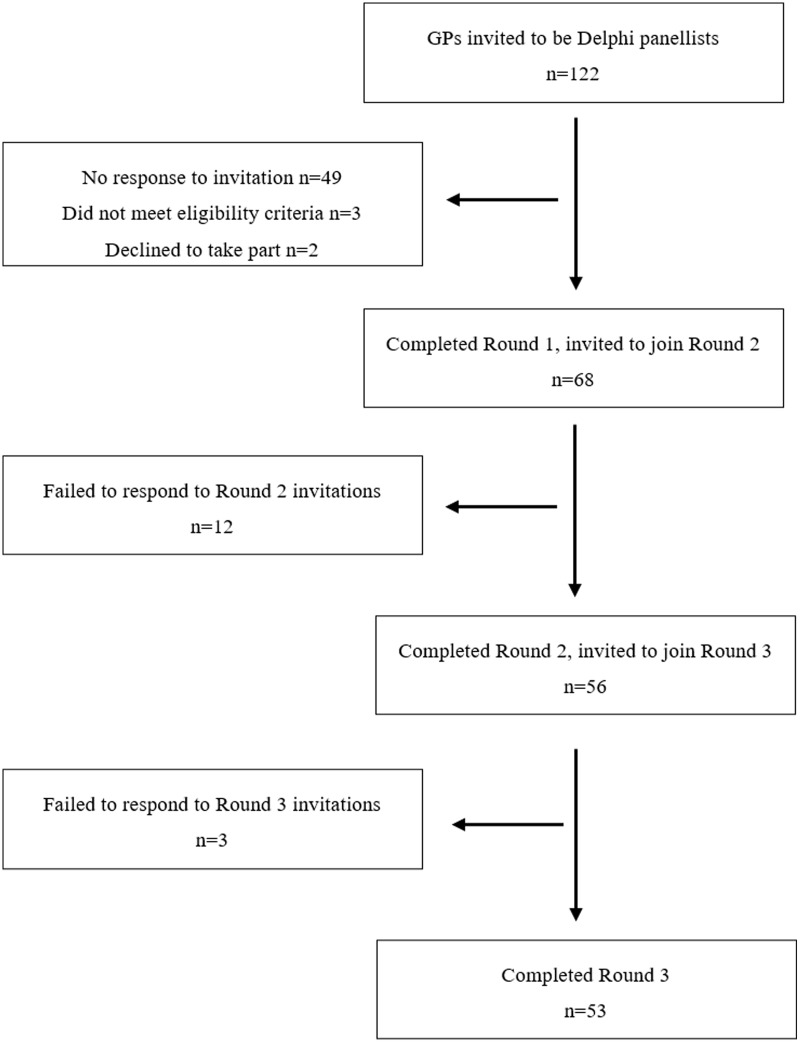
The number of GPs initially invited and the number of panellists for each of the Delphi rounds.

The Delphi study began in May 2020, and the process lasted four months. Figure 2 shows the number of GPs initially invited and the number of panellists for each of the three Delphi rounds.

### Delphi round response rates

In total, 105 GPs from 21 European countries, 5 panellists per country, were identified by their Local Leads and invited to participate. We invited 17 more GPs from the reserve lists of the countries with the lowest response rates. After two reminders, 71 GPs from 20 countries had completed the first round of the survey, 49 had not responded, and 2 had refused to participate. We excluded 3 responders because they did not meet the eligibility criteria. This meant that 68 eligible GPs completed Round 1 of the Delphi study and were invited to participate in Round 2.

Of those, 56 (82.3%) completed Round 2 and were invited to take part in Round 3.

In Round 3, 53 panellists (77.9% of those who had completed Round 1) completed the questionnaire.

The numbers of GPs involved, by country, at different stages of the process are presented in Appendix 2.

Table 2 shows the demographic data of the eligible GPs who completed Round 1.

**Table 2.** Demographic data of the 68 panellists who completed Round 1

Sex	
Male	32
Female	36
**Years of experience since graduation**	
0–9 years	16
10–19 years	18
20–29 years	25
30 years or more	9
**Academic status**	
Academic GP	36
Not academic GP	32
**Working area**	
Rural	14
Urban	36
Mixed	18

### Likert scale scores

Table 3 compares the mean Likert scale score SDs for all the statements combined across the three Delphi rounds, demonstrating an increase in the degree of consensus over the course of the rounds.

**Table 3.** Mean standard deviation (SD) of Likert scale scores for all the statements in the three Delphi rounds

	Mean SD of scores
Round 1	2.08
Round 2	1.90
Round 3	1.86

Twelve of the 52 statements satisfied the pre-defined selection criterion for how GPs believe they would be empowered to increase the number of early cancer diagnoses. These fell into four groups as presented in Table 4. All the 52 statements and their Round 3 scores, presented in descending order of their ‘mean statement score – SD’ value, are given in Appendix 3.

**Table 4.** The 12 statements satisfying the pre-defined Delphi selection criterion

Statements	Round 3 Likert scale score		
*GPs would be empowered to increase the number of early cancer diagnoses by…*	Mean	SD	Mean – SD
**Screening statements**			
…having a reliable system for screening patients who have a higher familial risk of cancer.	7.55	1.50	6.05
…having clear guidelines for cancer screening.	7.06	1.50	5.56
…having screening programmes that are more evidence-based.	7.08	1.57	5.51
…having electronic reminders for when individual patients need screening tests.	7.02	1.98	5.04
…providing more motivation for GPs to take part in screening.	6.58	1.55	5.04
**Links with secondary care statements**			
…having quicker, easier communication with secondary care.	7.42	1.47	5.94
…having better availability of rapid access pathways.	7.23	1.62	5.60
…having shorter waiting times for secondary care.	7.08	1.82	5.26
…being able to get quick advice from secondary care.	6.94	1.77	5.17
**GP working conditions and workload statements**			
…having a lower workload for GPs.	6.96	1.72	5.24
…having less bureaucracy for GPs.	7.19	1.99	5.20
**Information technology (IT) statements**			
…having better information technology (IT) to support communication and information transfer.	6.75	1.74	5.01

## Discussion

### Principal findings

In total, 53 panellists from 20 different countries across Europe completed the three rounds of the Delphi process. Twelve statements satisfied the pre-defined selection criterion for how GPs believe that they would be empowered when diagnosing patients who may have cancer. Five of the statements related to screening, and four to the patient and GP interface with secondary care. The other selected statements concerned IT and GPs’ working conditions. Statements relating to training, skills and working efficiency were not considered priority areas.

### Strengths and weaknesses

The Delphi statements were carefully developed and piloted by researchers from 23 European countries, most of whom were GPs, and therefore, the study was grounded in their clinical experience. The study had a broad spectrum of participating centres, with countries from each of the Central, Eastern, Northern, Southern and Western European geographical areas, providing variation in geography, socioeconomic and health systems, gate-keeping and referral systems, and levels of healthcare spending.

Although we did not aim to reach a representative sample of GPs in this study, most panellists were from the Local Leads’ own localities and over half of panellists were academic GPs, which may have led to sampling bias. The recruitment method used in this study resulted in variable response rates, leading to a risk of non-response bias. However, 53 GPs completed the Delphi process, 78% of those that had started it. While the findings are a consensus of GPs from 20 different European countries, some statements will have more relevance in some countries than others. While we defined a cut-off level for statement inclusion based on previous studies (Nyborg *et al.*, 2015) before we started to collect data, this choice of level was empirical.

The use of the Delphi methodology has some limitations in itself. There are no agreed standards on how to select the participants or how to interpret and analyse the results, and there are no universally agreed definitions of consensus (Fink-Hafner *et al.*, 2019). Methodological limitations include the difficulty of generalising the results to a wider population due to the sample size, limited views, the uneven spread of expertise among the panellists and panellists’ specific agendas. While the method encourages panellists to reconsider their views at each round in response to additional information, this flexibility can introduce bias, with participants altering their response to comply with what they view to be the majority opinion (Barrett, 2020).

### Comparison with existing literature

In line with our findings, Curry *et al.* (2003) have shown that factors related to cancer screening, as well as workload and links between primary and secondary care, are considered essential for enhancing doctors’ practice. In countries where cancer screening is not routinely available, our panellists think that this should be implemented, in order to empower them to make timely cancer diagnoses. Efforts to construct more reliable systems for cancer screening, an issue prioritised by our panellists, as well as incorporation of new knowledge into screening programmes and development of new technologies for cancer screening, are important for early cancer diagnosis (Loud and Murphy, 2017). While population-wide screening programmes for breast, cervical and colorectal cancers are active in a majority of European countries, these programmes typically function independently of primary care, ignoring opportunities for primary care to enhance the effectiveness of the programmes by promoting uptake, information provision and informed choice (Weller *et al.*, 2009). Evidence-based screening programmes support GPs’ empowerment by enabling them to avoid harms due to low-value or outdated screening programmes, and by conforming with populations’ interests (Parker *et al.*, 2017). Our study found that motivating GPs to take part in screening is considered an essential factor for empowerment. The most important motivating factors have been found to be the following: GP involvement in the planning of the screening programmes, receiving regular feedback on patients’ screening results, taking part in training courses, having a lower workload, and having financial incentives (Launoy *et al.*, 1993).

Our study indicated that having quicker and easier communication, shorter waiting times and getting prompt advice from secondary care are essential. Close links between primary and secondary health care are necessary for good quality patient care (Dinsdale *et al.*, 2020), since improved communication is associated with better health, better prevention, fewer hospital admissions and increased patient satisfaction; it also minimises treatment delays, additional workload, higher costs and reduced patient safety (Dinsdale *et al.*, 2020). GPs develop a sense of belonging and participation when establishing meaningful working relationships and having improved connectedness with specialists for their clinical work, which is essential for their clinical empowerment (Fulton *et al.*, 2016; Carrieri *et al.*, 2020). GPs’ early suspicion of cancer has been shown to improve outcomes if it reduces the time to diagnosis and treatment (Probst *et al.*, 2012). This supports our panel’s views that timely access to laboratory tests and other diagnostic processes, and use of rapid access pathways, empowers GPs in this field.

Our panel felt that better IT to support communication and information transfer is important: the exploitation of IT has been found to provide savings in time and effort, and, generally, a good experience with patients who nowadays are active in their care and use digital technologies in their health management (Mesko and Győrffy, 2019). Risk assessment tools and electronic reminders are also considered essential for assisting doctors’ efforts for early cancer diagnosis (Gomy and Diz, 2013; Printz, 2020).

Concerns about workload and bureaucracy, two areas that were prioritised by our panellists, have attracted attention to doctors’ working conditions, especially in relation to their health and risk of burnout (Scheepers *et al.*, 2020). A high level of bureaucracy takes GPs away from direct patient care (Schäfer, Berg and van der Groenewegen, 2020). Establishing a good balance between quantity and quality of time at work improves both quality of care and GPs’ job satisfaction (British Medical Association, 2020).

### Implications for research and practice

These study findings provide the basis for seeking actions and policies that will support GPs in their efforts in the timely diagnosis of cancer. European countries need establish reliable screening systems for cancer, where these do not already exist. Electronic Health Records (EHR) may be a valuable tool to aid detection of people with high familial risk, and this maps across to the panellists’ prioritisation of better IT. Their prioritisation of screening programmes that are more evidence-based implies that, in some countries at least, cancer screening programmes are based on unsound or outdated practices. There is a need to focus on the opportunities for primary care to enhance screening programmes, in particular risk-stratification within the population-based screening, risk-based early detection/screening and personalised screening.

The selected statements regarding the patient and GP interface with secondary care relate to speed of access: efficient channels for communication and advice, and shorter waits for specialist assessment. Health services need to assess their performance on these measures and prioritise faster access to secondary care for patients that could have cancer.

Qualitative studies are needed to describe exactly how GPs feel that these issues limit their empowerment. There is also a need for research involving GPs and other stakeholders to find out how the appropriate health policies can be promoted so that the most effective solutions are implemented.

While our Delphi panellists did not consider statements related to GPs’ training and skills to be a priority for empowering GPs in early cancer diagnosis, there is a need for more focused studies on this aspect.

## Conclusion

The role of Primary Health Care is crucial for the early diagnosis of cancer, as for many patients it is their first contact with the health system. Empowering GPs in their care of these patients is likely to lead to more timely cancer diagnoses.

The Delphi process identified twelve key statements representing factors that could empower GPs to increase the number of early cancer diagnoses. These statements relate to system factors, but do not include those relating to training, skills or working efficiency. The findings provide the basis for seeking actions and policies that will support GPs in their efforts to achieve a timely diagnosis of cancer.

## Supplementary material

For supplementary material accompanying this paper visit http://doi.org/10.1017/S1463423622000652. click here to view supplementary material

## Appendix 1. List of the 52 statements in 14 subject groups

### Continuing medical education (CME) for General Practitioners (GPs)

GPs would be empowered to increase the number of early cancer diagnoses by…

…having regular CME for GPs.

…having better quality CME for GPs.

…having more cancer-focused CME for GPs.

### GP skills & attitudes

GPs would be empowered to increase the number of early cancer diagnoses by…

…having better clinical skills.

…being dedicated to giving good quality care to patients who may have cancer.

…believing that they are competent to give good quality care to patients who may have cancer.

### GP knowledge

GPs would be empowered to increase the number of early cancer diagnoses by…

…GPs knowing more about when to investigate patients because of possible cancer.

…knowing how to get the right balance between over- and under-investigation of possible cancer.

…having better knowledge of atypical cancer symptoms and signs.

…having better knowledge of early cancer symptoms and signs.

…having better knowledge of, and access to, health indicators relating to cancer (prevalence, mortality, survival rate, etc.).

### GP performance

GPs would be empowered to increase the number of early cancer diagnoses by…

…being required to be competent in cancer diagnosis.

…having enough experience to be able to be confident in their care of patients who may have cancer.

### GP working conditions & workload

GPs would be empowered to increase the number of early cancer diagnoses by…

…having a lower workload for GPs.

…having longer consultations.

…having better working conditions for GPs.

…having less bureaucracy for GPs.

…having more personnel in primary care.

…having better payment for GPs.

…if it were easier for patients to get a GP appointment.

### Guidelines

GPs would be empowered to increase the number of early cancer diagnoses by…

…being more involved in designing cancer diagnosis guidelines and clinical pathways.

…having guidelines for non-specific symptoms that could be due to cancer.

…making more use of existing national or regional cancer guidelines.

### GP teams

GPs would be empowered to increase the number of early cancer diagnoses by…

…having better coordination within the Primary Healthcare (PHC) team.

…having more continuity of GP care (so that patients can usually see the same doctor each time).

…getting more feedback from their PHC colleagues.

### Health system

GPs would be empowered to increase the number of early cancer diagnoses by…

…having better financial support for early cancer diagnosis in primary care.

…having an understanding in the health system that, in order to diagnose more cancers early, they need to refer and investigate more patients.

…being under less pressure to reduce referrals.

…being more involved in planning and running primary care service.

…having an understanding in the health system that GPs’ ‘gut feelings’ are important.

### Information technology (IT)

GPs would be empowered to increase the number of early cancer diagnoses by…

…having electronic reminders for when individual patients need screening tests.

…having better IT to support communication and information transfer.

…having cancer diagnosis decision support in their IT systems.

### Links with secondary care

GPs would be empowered to increase the number of early cancer diagnoses by…

…having quicker, easier communication with secondary care.

…getting more feedback from secondary care.

…having shorter waiting times for secondary care.

…being able to get quick advice from secondary care.

### Pathways

GPs would be empowered to increase the number of early cancer diagnoses by…

…being more involved in designing rapid access pathways for suspected cancer.

…having better availability of rapid access pathways.

…having a simpler process for referral to a specialist.

### Patient issues

GPs would be empowered to increase the number of early cancer diagnoses by…

…having better public health measures to improve patients’ awareness of symptoms that could be due to cancer.

…having more reassurance that tests won’t be too expensive for their patients.

…being more trusted by their patients.

### Screening

GPs would be empowered to increase the number of early cancer diagnoses by…

…having a reliable system for screening patients who have a higher familial risk of cancer.

…having clear guidelines for cancer screening.

…having screening programmes that are more evidence-based.

…providing more motivation for GPs to take part in screening.

### Tests for cancer

GPs would be empowered to increase the number of early cancer diagnoses by…

…having easier GP access to tests for cancer.

…having shorter waiting times for tests for cancer.

…having special tests for cancer (CT scans or endoscopies, for example) available to GPs’ patients in the area where they live.

…being able to do diagnostic ultrasound in their practices.

## Appendix 2. Number of GPs involved, by country

**Table:**

Countries	Number of panellists		
	Initially Invited	Completed round 1	Completed round 3
Austria	6	5	4
Bulgaria	5	5	4
Croatia	6	3	3
England	5	1	1
Estonia	5	3	3
France	5	2	2
Greece	5	5	5
Hungary	7	2	2
Ireland	7	5	3
Israel	5	2	1
Italy	7	2	1
Latvia	6	4	3
Norway	6	3	2
Poland	6	5	3
Portugal	7	2	2
Romania	5	6	3
Scotland	5	0	0
Slovenia	7	3	3
Spain	6	3	2
Turkey	6	4	3
Ukraine	5	3	3
Total Responses	122	68	53

## Appendix 3. The 52 statements and their Round 3 scores in descending order of their ‘mean statement score – SD’ value

**Table:**

Statements	Round 3 Likert scale score		
*GPs would be empowered to increase the number of early cancer diagnoses by…*	Mean	SD	Mean – SD
**Statements satisfying the pre-defined Delphi selection criterion**			
…having a reliable system for screening patients who have a higher familial risk of cancer.	7.55	1.50	6.05
…having quicker, easier communication with secondary care.	7.42	1.47	5.94
…having better availability of rapid access pathways.	7.23	1.62	5.60
…having clear guidelines for cancer screening.	7.06	1.50	5.56
…having screening programmes that are more evidence-based.	7.08	1.57	5.51
…having shorter waiting times for secondary care.	7.08	1.82	5.26
…having a lower workload for GPs.	6.96	1.72	5.24
…having less bureaucracy for GPs.	7.19	1.99	5.20
…being able to get quick advice from secondary care.	6.94	1.77	5.17
…having electronic reminders for when individual patients need screening tests.	7.02	1.98	5.04
…providing more motivation for GPs to take part in screening.	6.58	1.55	5.04
…having better information technology (IT) to support communication and information transfer.	6.75	1.74	5.01
**Statements not satisfying the pre-defined Delphi selection criterion**			
…knowing how to get the right balance between over- and under-investigation of possible cancer.	6.70	1.74	4.96
…getting more feedback from secondary care.	6.83	1.90	4.93
…having longer consultations.	6.58	1.67	4.92
…having easier GP access to tests for cancer.	6.66	1.75	4.91
…having better public health measures to improve patients’ awareness of symptoms that could be due to cancer.	6.53	1.65	4.88
…having shorter waiting times for tests for cancer.	6.58	1.81	4.77
…having more personnel in primary care.	6.64	1.88	4.76
…GPs knowing more about when to investigate patients because of possible cancer.	6.36	1.63	4.73
…being more involved in designing rapid access pathways for suspected cancer.	6.34	1.66	4.68
…having better working conditions for GPs.	6.57	1.92	4.65
…having better knowledge of early cancer symptoms and signs.	6.23	1.67	4.55
…having an understanding in the health system that, in order to diagnose more cancers early, they need to refer and investigate more patients.	6.28	1.78	4.50
…having better financial support for early cancer diagnosis in primary care.	6.36	1.94	4.42
…having better knowledge of atypical cancer symptoms and signs.	6.11	1.71	4.41
…having more continuity of GP care (so that patients can usually see the same doctor each time).	6.23	1.89	4.34
…having a simpler process for referral to a specialist.	6.19	1.92	4.27
…having an understanding in the health system that GPs’ ‘gut feelings’ are important.	6.08	1.91	4.17
…having more cancer-focused CME for GPs.	5.91	1.82	4.08
…having better coordination within the Primary Healthcare (PHC) team.	5.85	1.78	4.07
…having cancer diagnosis decision support in their IT systems.	6.09	2.03	4.06
…having special tests for cancer (CT scans or endoscopies, for example) available to GPs’ patients in the area where they live.	6.09	2.08	4.02
…having guidelines for non-specific symptoms that could be due to cancer.	5.91	1.89	4.01
…getting more feedback from their PHC colleagues.	5.87	1.88	3.99
…being more involved in designing cancer diagnosis guidelines and clinical pathways.	5.91	1.96	3.94
…being dedicated to giving good quality care to patients who may have cancer.	5.89	1.98	3.91
…having enough experience to be able to be confident in their care of patients who may have cancer.	5.79	1.94	3.86
…having better knowledge of, and access to, health indicators relating to cancer (prevalence, mortality, survival rate etc).	5.62	1.84	3.78
…being more involved in planning and running primary care service.	5.96	2.18	3.78
…making more use of existing national or regional cancer guidelines.	5.66	1.91	3.75
…believing that they are competent to give good quality care to patients who may have cancer.	5.68	1.97	3.71
…having better clinical skills.	5.89	2.18	3.71
…having better quality CME for GPs.	5.83	2.16	3.67
…being able to do diagnostic ultrasound in their practices.	5.81	2.16	3.65
…having better payment for GPs.	5.91	2.29	3.62
…being more trusted by their patients.	5.53	1.93	3.60
…being under less pressure to reduce referrals.	5.68	2.09	3.59
…having regular CME for GPs.	5.57	2.03	3.53
…being required to be competent in cancer diagnosis.	5.51	2.02	3.49
…having more reassurance that tests won’t be too expensive for their patients.	5.28	2.10	3.19
…if it were easier for patients to get a GP appointment.	4.51	1.97	2.54
