# Supplementary material for: What factors empower general practitioners for early cancer diagnosis? A 20-country European Delphi Study
Source: Prim Health Care Res Dev. 2022 Nov 25;23:e76. doi: 10.1017/S1463423622000652 (PMC9706379; doi:10.1017/S1463423622000652)
Supplement: Supplementary file 1 [file phcsup.zip › S1463423622000652sup001.docx]

**Appendix 2 Number of GPs involved, by country**

| **Countries** | **Number of panellists** | | |
| --- | --- | --- | --- |
|  | Initially Invited | Completed round 1 | Completed round 3 |
| Austria | 6 | 5 | 4 |
| Bulgaria | 5 | 5 | 4 |
| Croatia | 6 | 3 | 3 |
| England | 5 | 1 | 1 |
| Estonia | 5 | 3 | 3 |
| France | 5 | 2 | 2 |
| Greece | 5 | 5 | 5 |
| Hungary | 7 | 2 | 2 |
| Ireland | 7 | 5 | 3 |
| Israel | 5 | 2 | 1 |
| Italy | 7 | 2 | 1 |
| Latvia | 6 | 4 | 3 |
| Norway | 6 | 3 | 2 |
| Poland | 6 | 5 | 3 |
| Portugal | 7 | 2 | 2 |
| Romania | 5 | 6 | 3 |
| Scotland | 5 | 0 | 0 |
| Slovenia | 7 | 3 | 3 |
| Spain | 6 | 3 | 2 |
| Turkey | 6 | 4 | 3 |
| Ukraine | 5 | 3 | 3 |
| Total Responses | 122 | 68 | 53 |
