# Supplementary material for: What factors empower general practitioners for early cancer diagnosis? A 20-country European Delphi Study
Source: Prim Health Care Res Dev. 2022 Nov 25;23:e76. doi: 10.1017/S1463423622000652 (PMC9706379; doi:10.1017/S1463423622000652)
Supplement: Supplementary file 1 [file phcsup.zip › S1463423622000652sup002.docx]

**Appendix 1 List of the 52 statements in 14 subject groups**

*Continuing medical education (CME) for General Practitioners (GPs)*

GPs would be empowered to increase the number of early cancer diagnoses by…

…having regular CME for GPs.

…having better quality CME for GPs.

…having more cancer-focused CME for GPs.

*GP skills & attitudes*

GPs would be empowered to increase the number of early cancer diagnoses by…

...having better clinical skills.

…being dedicated to giving good quality care to patients who may have cancer.

…believing that they are competent to give good quality care to patients who may have cancer.

*GP knowledge*

GPs would be empowered to increase the number of early cancer diagnoses by…

…GPs knowing more about when to investigate patients because of possible cancer.

…knowing how to get the right balance between over- and under-investigation of possible cancer.

…having better knowledge of atypical cancer symptoms and signs.

…having better knowledge of early cancer symptoms and signs.

…having better knowledge of, and access to, health indicators relating to cancer (prevalence, mortality, survival rate etc).

*GP performance*

GPs would be empowered to increase the number of early cancer diagnoses by…

…being required to be competent in cancer diagnosis.

…having enough experience to be able to be confident in their care of patients who may have cancer.

*GP working conditions & workload*

GPs would be empowered to increase the number of early cancer diagnoses by…

…having a lower workload for GPs.

…having longer consultations.

…having better working conditions for GPs.

…having less bureaucracy for GPs.

…having more personnel in primary care.

…having better payment for GPs.

…if it were easier for patients to get a GP appointment.

*Guidelines*

GPs would be empowered to increase the number of early cancer diagnoses by…

…being more involved in designing cancer diagnosis guidelines and clinical pathways.

…having guidelines for non-specific symptoms that could be due to cancer.

…making more use of existing national or regional cancer guidelines.

*GP teams*

GPs would be empowered to increase the number of early cancer diagnoses by…

…having better coordination within the Primary Healthcare (PHC) team.

…having more continuity of GP care (so that patients can usually see the same doctor each time).

…getting more feedback from their PHC colleagues.

*Health system*

GPs would be empowered to increase the number of early cancer diagnoses by…

...having better financial support for early cancer diagnosis in primary care.

…having an understanding in the health system that, in order to diagnose more cancers early, they need to refer and investigate more patients.

…being under less pressure to reduce referrals.

…being more involved in planning and running primary care service.

…having an understanding in the health system that GPs' “gut feelings” are important.

*Information technology (IT)*

GPs would be empowered to increase the number of early cancer diagnoses by…

…having electronic reminders for when individual patients need screening tests.

…having better IT to support communication and information transfer.

...having cancer diagnosis decision support in their IT systems.

*Links with secondary care*

GPs would be empowered to increase the number of early cancer diagnoses by…

…having quicker, easier communication with secondary care.

…getting more feedback from secondary care.

…having shorter waiting times for secondary care.

…being able to get quick advice from secondary care.

*Pathways*

GPs would be empowered to increase the number of early cancer diagnoses by…

…being more involved in designing rapid access pathways for suspected cancer.

…having better availability of rapid access pathways.

…having a simpler process for referral to a specialist.

*Patient issues*

GPs would be empowered to increase the number of early cancer diagnoses by…

…having better public health measures to improve patients' awareness of symptoms that could be due to cancer.

…having more reassurance that tests won't be too expensive for their patients.

...being more trusted by their patients.

*Screening*

GPs would be empowered to increase the number of early cancer diagnoses by…

…having a reliable system for screening patients who have a higher familial risk of cancer.

…having clear guidelines for cancer screening.

…having screening programmes that are more evidence-based.

…providing more motivation for GPs to take part in screening.

*Tests for cancer*

GPs would be empowered to increase the number of early cancer diagnoses by…

…having easier GP access to tests for cancer.

…having shorter waiting times for tests for cancer.

...having special tests for cancer (CT scans or endoscopies, for example) available to GPs’ patients in the area where they live.

…being able to do diagnostic ultrasound in their practices.
