# Supplementary material for: What factors empower general practitioners for early cancer diagnosis? A 20-country European Delphi Study
Source: Prim Health Care Res Dev. 2022 Nov 25;23:e76. doi: 10.1017/S1463423622000652 (PMC9706379; doi:10.1017/S1463423622000652)
Supplement: Supplementary file 1 [file phcsup.zip › S1463423622000652sup003.docx]

**Appendix 3 The 52 statements and their Round 3 scores in descending order of their “mean statement score - SD” value.**

| **Statements**  *GPs would be empowered to increase the number of early cancer diagnoses by…* | **Round 3 Likert  scale score** | | | | |
| --- | --- | --- | --- | --- | --- |
|  | **Mean** | **SD** | | **Mean ‑ SD** | |
| **Statements satisfying the pre-defined Delphi selection criterion**  …having a reliable system for screening patients who have a higher familial risk of cancer. | 7.55 | 1.50 | | 6.05 | |
| …having quicker, easier communication with secondary care. | 7.42 | 1.47 | | 5.94 | |
| …having better availability of rapid access pathways. | 7.23 | 1.62 | | 5.60 | |
| …having clear guidelines for cancer screening. | 7.06 | 1.50 | | 5.56 | |
| …having screening programmes that are more evidence-based. | 7.08 | 1.57 | | 5.51 | |
| …having shorter waiting times for secondary care. | 7.08 | 1.82 | | 5.26 | |
| …having a lower workload for GPs. | 6.96 | 1.72 | | 5.24 | |
| …having less bureaucracy for GPs. | 7.19 | 1.99 | | 5.20 | |
| …being able to get quick advice from secondary care. | 6.94 | 1.77 | | 5.17 | |
| …having electronic reminders for when individual patients need screening tests. | 7.02 | 1.98 | | 5.04 | |
| …providing more motivation for GPs to take part in screening. | 6.58 | 1.55 | | 5.04 | |
| …having better information technology (IT) to support communication and information transfer. | 6.75 | 1.74 | | 5.01 | |
| **Statements not satisfying the pre-defined Delphi selection criterion**  …knowing how to get the right balance between over- and under-investigation of possible cancer. | 6.70 | | 1.74 | | 4.96 |
| …getting more feedback from secondary care. | 6.83 | | 1.90 | | 4.93 |
| …having longer consultations. | 6.58 | | 1.67 | | 4.92 |
| …having easier GP access to tests for cancer. | 6.66 | | 1.75 | | 4.91 |
| …having better public health measures to improve patients' awareness of symptoms that could be due to cancer. | 6.53 | | 1.65 | | 4.88 |
| …having shorter waiting times for tests for cancer. | 6.58 | | 1.81 | | 4.77 |
| …having more personnel in primary care. | 6.64 | | 1.88 | | 4.76 |
| …GPs knowing more about when to investigate patients because of possible cancer. | 6.36 | | 1.63 | | 4.73 |
| …being more involved in designing rapid access pathways for suspected cancer. | 6.34 | | 1.66 | | 4.68 |
| …having better working conditions for GPs. | 6.57 | | 1.92 | | 4.65 |
| …having better knowledge of early cancer symptoms and signs. | 6.23 | | 1.67 | | 4.55 |
| …having an understanding in the health system that, in order to diagnose more cancers early, they need to refer and investigate more patients. | 6.28 | | 1.78 | | 4.50 |
| ...having better financial support for early cancer diagnosis in primary care. | 6.36 | | 1.94 | | 4.42 |
| …having better knowledge of atypical cancer symptoms and signs. | 6.11 | | 1.71 | | 4.41 |
| …having more continuity of GP care (so that patients can usually see the same doctor each time). | 6.23 | | 1.89 | | 4.34 |
| …having a simpler process for referral to a specialist. | 6.19 | | 1.92 | | 4.27 |
| …having an understanding in the health system that GPs' “gut feelings” are important. | 6.08 | | 1.91 | | 4.17 |
| …having more cancer-focused CME for GPs. | 5.91 | | 1.82 | | 4.08 |
| …having better coordination within the Primary Healthcare (PHC) team. | 5.85 | | 1.78 | | 4.07 |
| ...having cancer diagnosis decision support in their IT systems. | 6.09 | | 2.03 | | 4.06 |
| ...having special tests for cancer (CT scans or endoscopies, for example) available to GPs’ patients in the area where they live. | 6.09 | | 2.08 | | 4.02 |
| …having guidelines for non-specific symptoms that could be due to cancer. | 5.91 | | 1.89 | | 4.01 |
| …getting more feedback from their PHC colleagues. | 5.87 | | 1.88 | | 3.99 |
| …being more involved in designing cancer diagnosis guidelines and clinical pathways. | 5.91 | | 1.96 | | 3.94 |
| …being dedicated to giving good quality care to patients who may have cancer. | 5.89 | | 1.98 | | 3.91 |
| …having enough experience to be able to be confident in their care of patients who may have cancer. | 5.79 | | 1.94 | | 3.86 |
| …having better knowledge of, and access to, health indicators relating to cancer (prevalence, mortality, survival rate etc). | 5.62 | | 1.84 | | 3.78 |
| …being more involved in planning and running primary care service. | 5.96 | | 2.18 | | 3.78 |
| …making more use of existing national or regional cancer guidelines. | 5.66 | | 1.91 | | 3.75 |
| …believing that they are competent to give good quality care to patients who may have cancer. | 5.68 | | 1.97 | | 3.71 |
| ...having better clinical skills. | 5.89 | | 2.18 | | 3.71 |
| …having better quality CME for GPs. | 5.83 | | 2.16 | | 3.67 |
| …being able to do diagnostic ultrasound in their practices. | 5.81 | | 2.16 | | 3.65 |
| …having better payment for GPs. | 5.91 | | 2.29 | | 3.62 |
| ...being more trusted by their patients. | 5.53 | | 1.93 | | 3.60 |
| …being under less pressure to reduce referrals. | 5.68 | | 2.09 | | 3.59 |
| …having regular CME for GPs. | 5.57 | | 2.03 | | 3.53 |
| …being required to be competent in cancer diagnosis. | 5.51 | | 2.02 | | 3.49 |
| …having more reassurance that tests won't be too expensive for their patients. | 5.28 | | 2.10 | | 3.19 |
| …if it were easier for patients to get a GP appointment. | 4.51 | | 1.97 | | 2.54 |
